# AWclust: point-and-click software for non-parametric population structure analysis

**DOI:** 10.1186/1471-2105-9-77

**Published:** 2008-01-31

**Authors:** Xiaoyi Gao, Joshua D Starmer

**Affiliations:** 1 Miami Institute for Human Genomics, University of Miami Miller School of Medicine, Miami, FL 33136, USA; 2 Department of Genetics, University of North Carolina at Chapel Hill, Chapel Hill, NC 27599, USA; 3 Curriculum in Toxicology, University of North Carolina at Chapel Hill, Chapel Hill, NC 27599, USA

## Abstract

**Background:**

Population structure analysis is important to genetic association studies and evolutionary investigations. Parametric approaches, e.g. STRUCTURE and L-POP, usually assume Hardy-Weinberg equilibrium (HWE) and linkage equilibrium among loci in sample population individuals. However, the assumptions may not hold and allele frequency estimation may not be accurate in some data sets. The improved version of STRUCTURE (version 2.1) can incorporate linkage information among loci but is still sensitive to high background linkage disequilibrium. Nowadays, large-scale single nucleotide polymorphisms (SNPs) are becoming popular in genetic studies. Therefore, it is imperative to have software that makes full use of these genetic data to generate inference even when model assumptions do not hold or allele frequency estimation suffers from high variation.

**Results:**

We have developed point-and-click software for non-parametric population structure analysis distributed as an R package. The software takes advantage of the large number of SNPs available to categorize individuals into ethnically similar clusters and it does not require assumptions about population models. Nor does it estimate allele frequencies. Moreover, this software can also infer the optimal number of populations.

**Conclusion:**

Our software tool employs non-parametric approaches to assign individuals to clusters using SNPs. It provides efficient computation and an intuitive way for researchers to explore ethnic relationships among individuals. It can be complementary to parametric approaches in population structure analysis.

## Background

Population structure analysis is important to genetic association studies [1-4] and evolutionary investigations [5-9]. Many statistical methods have been proposed to infer population structure and to assign individuals to ethnically similar clusters using multilocus genotype data, among which there are two major categories: parametric and non-parametric approaches.

Parametric approaches usually need to estimate population parameters such as allele frequencies and genotype frequencies and calculate likelihood, assuming Hardy-Weinberg equilibrium (HWE) and linkage equilibrium (LE) among loci for each population [10,11]. Two representative programs for parametric approaches are: STRUCTURE, a Bayesian method which uses a Markov chain Monte Carlo (MCMC) algorithm based on the Gibbs sampler algorithm [10], and L-POP, a frequentist method which uses the Expectation-Maximization (EM) algorithm [11]. In the extended version of STRUCTURE (version 2.1), the program can account for loose linkage between loci, but not high background linkage disequilibrium (LD) [12,13]. High background LD increases the chance of spurious clusters [13]. There are many other parametric Bayesian methods [14-20] and frequentist methods [21,22], which require similar or more complicated model assumptions. Two major challenges for the parametric approaches are the accuracy of allele frequencies estimates with small sample sizes, and the model assumptions that may not hold for some data sets. Moreover, assumptions of LE or loosely linked loci put a restriction on the number of genome-wide SNP loci that can be used.

In contrast to parametric approaches, non-parametric approaches do not rely on model assumptions about the properties of the sub-populations, nor do they require allele frequency estimates. In situations where parametric model assumptions can not be verified, or there is only a limited number of individuals from a single sub-population, non-parametric methods are more powerful for inference. However, when the model assumptions do hold and allele frequencies can be accurately estimated, then parametric methods provide more information. Thus, the two approaches are complementary in that one method is stronger where the other is weaker.

As stated by Liu and Zhao [23], non-parametric methods use a two-stage design. They start by calculating pair-wise distances [6,7,9], or some other form of dimension reduction, e.g. singular value decomposition (SVD) [23], and then rely on statistical clustering methods, e.g. neighbor joining (NJ) [6,7], K-means method [23], principal coordinates analysis (PCoA) [9,24] or multidimensional scaling (MDS) [25,26], to separate individuals. Recently, Gao and Starmer proposed a non-parametric method for population structure analysis and showed its advantages when genome-wide SNPs are available [27]. Liu and Zhao also proposed a non-parametric approach [23], but it requires missing genotypes be imputed explicitly and the software is not widely available. In recent publications, researchers tend to use both parametric and non-parametric approaches in their reports [24,25,28].

Since its publication in 2000, the freely available program STRUCTURE has become quite popular and dominated population structure analysis, while the non-parametric methods have not received much attention. However, with the vast amount of genotype data available, non-parametric approaches may be preferred because of their robustness to model assumptions and fast calculation. Recently, it was shown in an empirical study that non-parametric methods can give accurate results in fine-scale population structure detection and even separated Chinese and Japanese individuals using genome-wide random SNPs [27]. The separation of Chinese and Japanese individuals was also observed by Purcell et al. using MDS [26].

R is a convenient fast growing statistical computing environment with considerable popularity in the research community. It is freely available on a wide range of platforms, comes with implementations of many standard statistical methods, and can be easily extended through packages. We borrowed the strength of R and developed an add-on package that specifically focused on non-parametric population structure analysis. The motivation behind the package is to make recent developments in non-parametric population structure analysis available to researchers with an easy to use and intuitive graphical interface.

## Implementation

We have developed point-and-click software for non-parametric population structure exploration. The program was written in R and Tk and can be installed as an R package. The package is named AWclust (Allele sharing distance and Ward's minimum variance hierarchical clustering). The major modules enclosed are: allele sharing distance (ASD) calculation, MDS 2D plot, MDS 3D plot, hierarchical plot by Ward's minimum variance algorithm, gap statistic calculation for inferring the optimal number of clusters and saving the clustered results.

## Results

We automated the non-parametric population structure analysis procedures and packed all the routine steps in an intuitive graphical interface. The software can save researchers' time in data exploration. The outputs from AWclust are ready for publication and further analysis. It is distributed as R installation packages available for all popular operating systems: MS Windows, Mac OS X and Linux/Unix. The software comes with two example data sets, is fully documented, and includes a tutorial to help users become familiar with how to use it.

**Figure 1:**
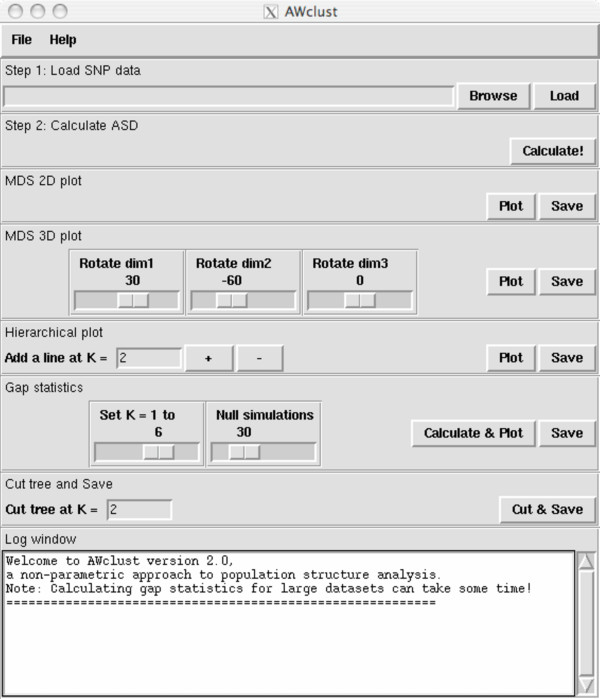
The AWclust interface.

After installing and loading the AWclust package, users are presented with a GUI interface (see Figure 1). To experiment with the program, users can load one of the two sample data sets: hapmap500 or perlegen500. The hapmap500 dataset contains 500 genome-wide random SNPs from 209 unrelated individuals from the HapMap project, specifically 60 Yoruba from Ibadan, Nigeria (YRI), 60 CEPH Utah residents with ancestry from northern and western Europe (CEU), 45 Han Chinese from Beijing, China (CHB), and 44 Japanese from Tokyo, Japan (JPT). The perlegen500 dataset contains 500 genome-wide random SNPs from 71 unrelated individuals from the Perlegen project, including 23 African Americans (AA), 24 European Americans (EA) and 24 Han Chinese (HC). In each dataset, the SNP information is encoded as numeric values (i.e. 0, 1, or 2) to represent the number of variant SNP alleles in genotypes, and -1 is used to represent missing values.

After loading a SNP data set, the next step is to calculate the allele sharing distance (ASD) matrix. Once this is done the user can perform non-parametric exploration with the SNP data set. The user can generate multidimensional scaling (MDS) 2D/3D plots to get a general idea of how the data clusters and to detect any outliers in the dataset. MDS is a statistical technique for allowing differences and similarities to be visualized. The differences are represented by distances between points on a graph. Elements in the ASD matrix that are close together will tend to cluster together.

If the MDS plot does not reveal any outliers in the dataset, then it makes sense to create a hierarchical plot of the data. For large datasets, the screen size and resolution may cause the IDs for the individuals to become difficult to read, however, this problem can be solved by saving and viewing the PDF output separately. The hierarchical plot can help users identify clusters and general relationships among individuals. The cluster tree can be cut at any level of similarity according to researchers' need and be saved to a text file.

One of the important features of AWclust is that it will calculate the gap statistic, a method for estimating the number of clusters in a data set [29]. It compares the pooled within-cluster sum of squares with its expectation from a null reference distribution. The precision of this method requires multiple simulations from the null reference distribution and thus, can be computationally intensive. For example, a dataset with 209 individuals, each with 1000 SNPs and running 60 simulations (K = 1 to 6) takes slightly longer than two minutes to run on an Intel Core2, 2.4 GHz CPU with 2 GB of RAM. The data points are then plotted for a range of cluster sizes and the optimal size maximizes the distance between the observed and expected pooled within-cluster sum of squares. Information from MDS and hierarchical plots may also help interpret of the gap statistic plots.

When using AWclust on datasets other than the provided samples, it is important to note that the more closely related the subpopulations are, the larger the number of genome-wide random SNP loci needed for good separation. Fortunately, AWclust can quickly process large datasets. For the number of SNP loci required for major human populations, users can refer the empirical studies by Gao and Starmer [27].

## Discussion

Genome-wide SNPs can be easily obtained using Affymetrix or Illumina chips and thus, making full use of the vast amount of genetic markers available is an open issue for parametric approaches since the LE assumption does not hold when SNPs are densely genotyped. Without complicated LD modeling, there is a bottleneck for the amount of data that can be used by parametric approaches. Moreover, the allele frequency estimation in parametric approaches suffers from high variation if the number of individuals for a sub-population is extremely small, e.g. less than ten, and this leads to questionable results.

Besides STRUCTURE, there are several other parametric software packages that are compared by Wu et al. [22], such as PARTITION [15], BAPS 2 [19], GENELAND [20] and PSMIX [22]. However, all parametric approaches suffer similar limitations because they all require verification of their model assumptions. When they are not justified, and for densely genotyped SNPs or when there is only a limited number of individuals from a single sub-population this is likely to be the case, non-parametric approaches provide better results.

Recently researchers have began to use both parametric and non-parametric approaches to aid population structure analysis [24,25,28]. However, non-parametric approaches have been limited to the utilization of NJ, K-means method, PCoA or MDS, all of which can not give the optimal number of clusters objectively. Furthermore, to our knowledge, we are not aware of an easy to use software package for non-parametric population structure analysis. Therefore, it is imperative to promote a non-parametric software package that automates routine steps and can take advantage of the vast amount of SNP markers available while inferring the optimal *K *objectively.

A limitation for the AWclust software is that it does not estimate the proportion of genome that belongs to each sub-population, which would necessarily require allele frequency estimation and many other assumptions about HWE and LD, while AWclust assigns each individual to one and only one cluster.  Furthermore, AWclust only handles SNPs, currently the most popular genetic marker. Also, we did not aim to cover as many features in population genetics analysis as possible, like Arlequin does [30] since we did not want to replicate functions available in other software. There is also algorithm/software targeting on admixture mapping, i.e. ADMIXMAP [14,16,17] and ANCESTRYMAP [31], which requires more complicated model assumptions and is out of the scope of non-parametric methods. AWclust performs classification rather than test statistic adjustment as genomic control [32-34] and EigenStrat [35] do. However, investigators can conduct further analysis conditional on the cluster information provided by AWclust.

A general challenge in population structure analysis is to infer the optimal number of populations, *K*, and this is no different for the AWclust software. Before using the gap statistic to infer the optimal *K*, we suggest to plot the data using MDS first in addition to the hierarchical plot. In our experience, hierarchical plots give general information about the clusters embedded in the data, while MDS is suitable for outlier detection in exploration of individual relationships. To give an extreme situation, if there is just one individual for a particular sub-population, it is unlikely this individual can form a stand-alone cluster among all of the hierarchical clusters. Therefore, it is better to take outlier individuals out before applying the hierarchical plot and the gap statistic. The optimal *K *can also be explained in combination with other prior information about populations in the data sets and experience of the field.

## Conclusion

In summary, we have developed new software, AWclust, for non-parametric population structure exploration. The software does not require HWE and LE for sample individuals. Nor does it need to estimate allele frequency. Most importantly, it can identify the optimal number of populations objectively. AWclust provides a user friendly GUI interface and does not require any prior programming skill from users. This non-parametric software is complementary to the parametric population structure programs because it is useful when HWE and LE can not be assumed to hold and when the accuracy of allele frequency estimation is questionable.

## Availability and requirements

**Project name: **AWclust

**Project home page: **

**Online users' manual: **

**Operating system(s): **Platform independent

**Programming language: **R, Tk

**Other requirements: **R 2.5 or higher (with Tk Widgets)

**License: **GPL

**Any restrictions to use by non-academics: **none

## Authors' contributions

XG designed and programmed the software. JS participated in programming and wrote the manual. XG and JS co-drafted the manuscript. Both authors read and approved the final manuscript.
